# Postoperative serum myoglobin as a predictor of early allograft dysfunction after liver transplantation

**DOI:** 10.3389/fsurg.2022.1026586

**Published:** 2022-10-12

**Authors:** Jin Zhang, Yuzhen Han, Shuhao Ke, Rongyue Gao, Xiaocui Shi, Song Zhao, Pan You, Huimiao Jia, Qi Ding, Yue Zheng, Wenxiong Li, Lifeng Huang

**Affiliations:** ^1^ Department of Surgical Intensive Care Unit, Beijing Chaoyang Hospital Affiliated to Capital Medical University, Beijing, China; ^2^ Department of Intensive Care Unit, Chengde Medical University, China

**Keywords:** myoglobin, early allograft dysfunction, liver transplantation, predictor, complication

## Abstract

**Background:**

Early allograft dysfunction (EAD) is a common postliver transplant complication that has been associated with graft failure and risk for poor prognosis. There are many risk factors for the incidence of EAD after liver transplantation (LT). This study investigated whether elevated postoperative myoglobin (Mb) increases the incidence of EAD in liver transplanted recipients.

**Methods:**

A total of 150 adult recipients who measured Mb within 3 days after liver transplantation between June 2019 and June 2021 were evaluated. Then, all patients were divided into two groups: the EAD group and the non-EAD group. Univariate and multivariate logistic regression analyses were performed, and receiver operating characteristic curves (ROCs) were constructed.

**Results:**

The incidence of EAD was 53 out of 150 patients (35.3%) in our study. Based on the multivariate logistic analysis, the risk of EAD increased with elevated postoperative Mb (OR = 1.001, 95% CI 1.000–1.001, *P *= 0.002). The Mb AUC was 0.657, and it was 0.695 when combined with PCT. When the subgroup analysis was conducted, the AUC of serum Mb prediction was better in patients whose preoperative model for end-stage liver disease score  ≤ 15 or operative time ≥ 10 h (AUC = 0.751, 0.758, respectively, or 0.760, 0.800 when combined with PCT).

**Conclusion:**

Elevated Mb significantly increased the risk of postoperative EAD, suggesting that postoperative Mb may be a novel predictor of EAD after liver transplantation.

The study was registered in the Chinese Clinical Trial Registry (Registration number: ChiCTR2100044257, URL: http://www.chictr.org.cn).

## Introduction

The most effective current treatment for end-stage liver disease (ESLD) is liver transplantation (LT) (1). However, due to the shortage of liver allografts, more extended standard allografts have been used in the clinic (2). There are still many complications associated with LT, such as early allograft dysfunction (EAD), which represents higher mortality and graft failure (3). A few patients with EAD could gradually develop into primary graft nonfunction, which is the most serious complication after orthotropic liver transplantation and often requires surgery again, with high clinical mortality (4, 5). There are many mechanisms of EAD, among which ischemia-reperfusion injury (IRI) remains the most likely (6). There are also many risk factors for the incidence of EAD, such as donor body mass index (BMI), uric acid, postoperative hepatic artery resistance index, and the C-reactive protein-to-albumin ratio, which have been reported in previous studies (5, 7–9).

Myoglobin (Mb) has been widely studied in previous studies. It is a small pigment protein produced by combining globin and heme-iron (10). Mb, as an O_2_ storage depot, is present in smooth muscle cells and cardiac myocytes (11). Due to the prolonged immobilization or certain surgical positions such as dorsal lithotomy and knee-chest position, another possibility of IRI, large amounts of myoglobin are released into the blood, which may result in organ failure (12). Myoglobin is a biomarker for myocardial infarction known to us (13). Hypermyoglobin may also result in acute kidney injury (AKI), as described in our previous study (14). In addition, among patients with rhabdomyolysis, the level of myoglobin was a strong predictor of AKI (15). Additionally, some studies found that postoperative serum myoglobin after cardiac surgery was associated with poor outcomes (16). In our center, we interestingly found that the level of serum myoglobin was significantly elevated in patients with EAD after LT. However, no previous studies have shown the correlation between myoglobin and the occurrence of EAD after LT.

Our study aims to provide the first description of the level of serum myoglobin on postoperative day 0 (POD0) in patients after LT. We further describe the tendency of serum Mb within 3 days after LT and the difference in Mb between patients with EAD and those without EAD.

## Materials and methods

### Study population

**Figure 1:**
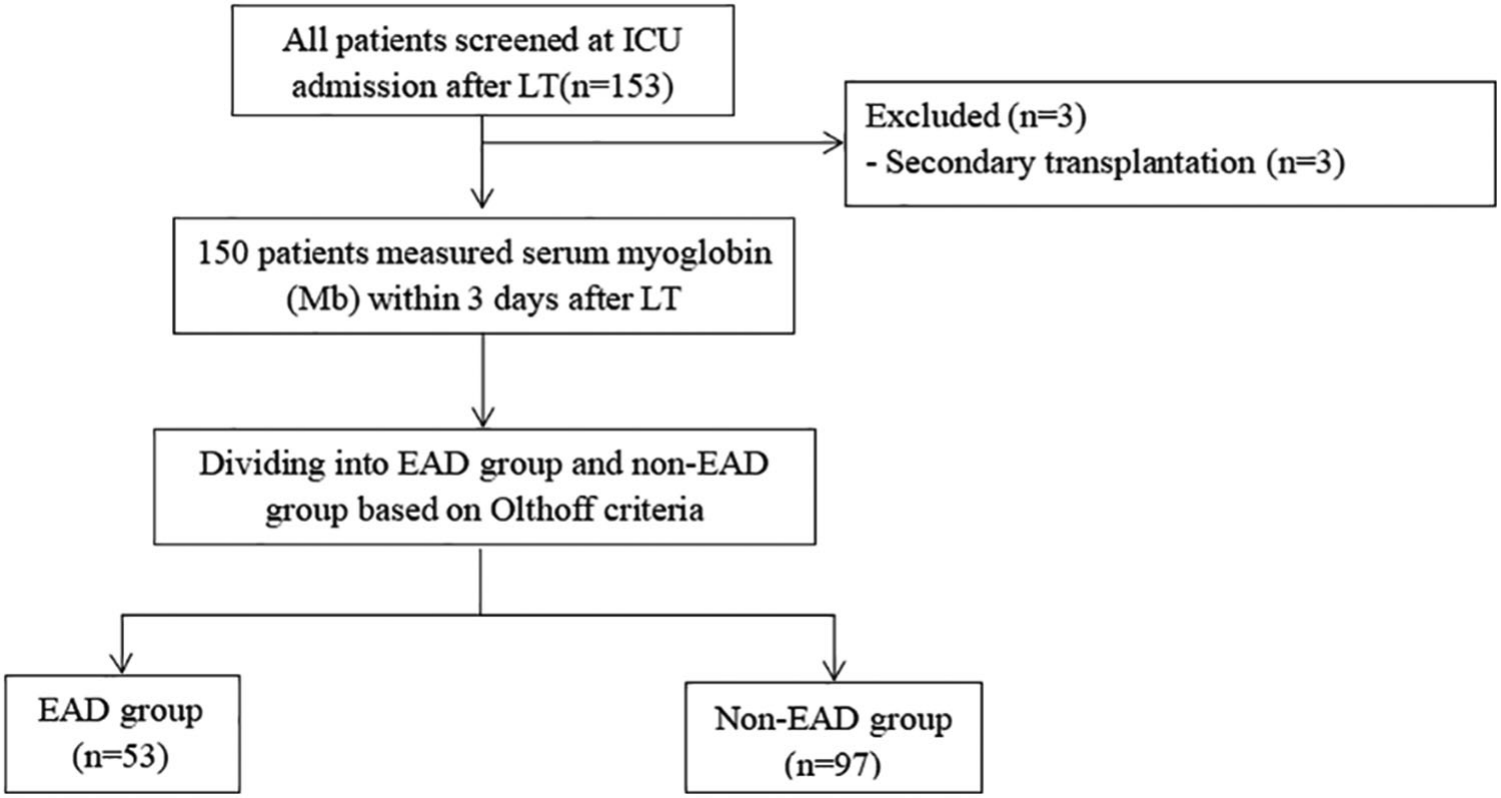
Study flow chart. Three patients with secondary transplantation were excluded. A total of 150 patients were included for analysis. ICU, intensive care unit; LT, liver transplantation; POD, postoperative day; Mb, myoglobin.

All patients who underwent orthotropic liver transplantation (OLT) were prospectively collected from June 2019 to June 2021 in the surgical intensive care unit (ICU) of Beijing Chao-yang Hospital. The informed consent had been obtained. The research program was approved by the Institutional Review Board (Beijing Chao-yang Hospital Affiliated to Capital Medical University, approval number: 2021-55) and was performed in compliance with the declaration of Helsinki. Also, neither piggyback nor venovenous bypass was used in all patients. The grafts were all from donation after brain death (DBD). The included criteria are as follows: (1) age ≥18 years and (2) admission to ICU after LT. Patients were excluded when they met any of the following criteria: (1) secondary liver transplantation; (2) age less than 18 years old; and (3) refused the informed consent. According to the incidence of EAD after LT which was reported in previous study 46.4%, a sample of 159 patients was required at a significance level of *a* = 0.05, with a power of 80% and allowing for 10% missing data. Finally, a total of 150 patients were included for analysis in our study, as shown in Figure 1.

### Clinical data collection

Demographic data including age, sex, BMI, the model for end-stage liver disease (MELD) score, Child–Pugh classification, and comorbidities (history of Ascites, encephalopathy, diabetes mellitus, etc.) were recorded as baseline data. Preoperative data such as white blood cell count (WBC), hemoglobin, platelet count, albumin, aspartate aminotransferase (AST) and alanine aminotransferase (ALT), total bilirubin (TB), direct bilirubin (DB), ammonia, international normalized ratio (INR), prothrombin time (PT), prothrombin activity (PA), and serum creatinine were collected from the latest data before surgery. These data were measured again upon admission to ICU immediately. In addition, laboratory data including AST, ALT, TB, and INR were measured within postoperative 7 days. Also, for all patients, serum myoglobin was measured at the time of admission to ICU and on POD1, POD2, and POD3. However, due to the purpose of predictive utility for EAD, we used the level of Mb on POD0 to explore the value of Mb to predict EAD. Furthermore, we obtained the Sequential Organ Failure Assessment (SOFA) score, the Acute Physiology Chronic Health Disease Classification System II (APACH II) score, and the use of vasopressor on POD0. We also evaluated whether sepsis was diagnosed upon admission to ICU. Intraoperative data including duration of surgery, blood loss, fluid balance, duration of inferior vena cava interruption, intraoperative urine output, cold ischemia time, and warm ischemia time were also obtained from the electronic medical record system.

### Definition of outcomes

The primary outcome of this study was the incidence of EAD within 7 days after surgery. Early allograft dysfunction was defined according to Olthoff et al., which met one or more of the following items: (1) serum levels of ALT or AST > 2000 U/L within the first week; (2) TB ≥ 10 mg/dl on postoperative day 7; and (3) INR ≥ 1.6 on postoperative day 7 (17). Patients who met one or more of the above criteria were included in the EAD group, and others were included in the non-EAD group. Secondary outcomes included AKI, the length of ICU and hospital stay, and 28-day overall mortality. AKI was defined as per the following criteria, according to Kidney Disease: Improving Global Outcome (KDIGO) criteria: an increase in serum creatinine by ≥26.5 μmol/L (≥3 mg/dl) within 48 h or an increase in serum creatinine to ≥1.5 times baseline, which is known or presumed to have occurred within the first 7 postoperative days (Supplementary Table S1) (18).

### Statistical analysis

All data were analyzed by SPSS statistical software version 26.0 (IBM, Chicago, IL, USA) and GraphPad Prism 8.3.0 (Graphpad Software Inc., San Diego, CA, USA). Our data were presented as the mean ± standard deviation (SD) and the median (interquartile range, IQR) for continuous variables or frequency (percentages) for categorical variables. The baseline characteristics, perioperative parameters, and outcomes were compared using the Student *t-*test, Mann–Whitney *U* test, chi-square test, or Fisher exact test as appropriate. In addition, univariate and multivariate logistic regression analyses were also performed to examine the effect of the level of Mb on POD0 on primary and secondary outcomes. We used multiple imputations to account for missing data. The receiver operating characteristic (ROC) curve analysis was performed to evaluate the predictive value of serum Mb on POD0 for EAD. Associations were expressed as odds ratio (OR) and 95% confidence interval (95% CI). Two-sided *P* values less than 0.05 were considered statistically significant.

## Results

### Comparison of baseline characteristics

Excluding 3 patients who underwent the second LT, a total of 150 patients were initially recruited for the current study, including 127 (84.7%) men and 23 (15.3%) women (Figure 1). The mean ± SD of age and BMI were 52.6 ± 9.4 years and 23.7 ± 3.6 kg/m^2^. There were 53 (35.3%) patients occurring with EAD after LT. Overall, the etiology of LT is as follows: 44.0% liver tumor, 25.3% viral diseases, 10.7% alcohol-related, 7.3% biliary cirrhosis, and 12.7% other liver diseases. All patients received liver grafts from donation after brain death (DBD). There was no statistical difference in demographics between the two groups (*P *> 0.05), as shown in Table 1.

**Table 1:** Characteristics of EAD and non-EAD patients.

Groups	All (*n* = 150)	EAD (*n* = 53)	Non-EAD (*n* = 97)	*P* value
Characteristics				
Age (years)	52.6 ± 9.4	52.4 ± 9.3	52.6 ± 9.4	0.871
Female [*n* (%)]	23 (15.3)	7 (13.2)	16 (16.5)	0.593
BMI (kg/m^2^)	23.7 ± 3.6	23.1 ± 3.9	24.0 ± 3.4	0.188
MELD score	13.0 (9.0, 21.0)	16.5 (9.0, 21.0)	15.7 (9.0, 20.0)	0.833
MELD-Na score	12.0 (9.0, 20.9)	15.9 (8.0, 21.0)	15.0 (9.0, 18.3)	0.689
Child–Pugh classification				
Class A or B [*n* (%)]	77 (51.3)	29 (54.7)	48 (49.5)	0.540
Class C [*n* (%)]	73 (48.7)	24 (45.3)	49 (50.5)	
Etiologies of liver disease				
Viral diseases [*n* (%)]	38 (25.3)	12 (22.6)	26 (26.8)	0.575
Liver tumor [*n* (%)]	66 (44.0)	24 (45.3)	42 (43.3)	0.815
Alcohol-related [*n* (%)]	16 (10.7)	6 (11.3)	10 (10.3)	0.848
Biliary cirrhosis [*n* (%)]	11 (7.3)	3 (5.7)	8 (8.2)	0.561
Others [*n* (%)]	19 (12.7)	8 (15.1)	11 (11.3)	0.509
Comorbidities				
Diabetes mellitus [*n* (%)]	27 (18.0)	8 (15.1)	19 (19.6)	0.494
Hypertension [*n* (%)]	34 (22.7)	11 (20.8)	23 (23.7)	0.679
Coronary disease [*n* (%)]	6 (4.0)	2 (3.8)	4 (4.1)	>0.999
Complications related to the liver				
Ascites [*n* (%)]	23 (15.3)	7 (13.2)	16 (16.5)	0.593
Hepatic encephalopathy [*n* (%)]	12 (8.0)	5 (9.4)	7 (7.2)	0.632
Infection [*n* (%)]	19 (12.7)	8 (15.1)	11 (11.3)	0.509

Values were expressed as mean ± SD, mean (IQR), or *n* (%).SD, standard deviation; IQR, interquartile range; EAD, early allograft dysfunction; BMI, body mass index; MELD, model for end-stage liver disease.

### Comparison of perioperative variables

The comparison of baseline characteristics between the two groups is listed in Table 2. Patients in the EAD group had higher WBC counts (3.72 [2.79, 6.60] vs. 3.34 [2.18, 5.00], *P *= 0.021) than those in the non-EAD group. In terms of intraoperative variables, we could find that patients with EAD experienced a longer duration of surgery, even though there was no difference between the two groups (*P *= 0.256). Significantly, the mean intraoperative urine output in the EAD group patients was lower than that in the non-EAD group patients (168.8 [103.8, 225.3] vs. 187.3 [124.9, 268.9], *P *= 0.050). Postoperative data including SOFA score, APACH II score, lactate, WBC, PLT, PA, Mb, and PCT were obtained upon admission to ICU immediately. There was also a statistical significance in the level of postoperative lactate (2.1 [1.5, 4.1] vs. 1.6 [1.1, 2.6], *P *= 0.011), APACH II score (15.9 ± 8.0 vs. 14.2 ± 6.0, *P *= 0.023), PA (33.1 [23.6, 42.1] vs. 39.9 [31.4, 47.6], *P *= 0.002), and PCT (30.9 [8.5, 82.5] vs. 14.2 [4.7, 28.6], *P *= 0.007) between the two groups. Patients with EAD were more likely to have a prolonged ICU stay (5.6 [3.3, 10.4] vs. 4.2 [2.7, 5.7], *P *= 0.003). Moreover, of the 150 patients, 11 (7.4%) patients died, 7 (13.2%) in the EAD group and 4 (4.1%) in the non-EAD group (*P *= 0.041). No significant difference was observed in other variables between the two groups.

**Table 2:** Comparison of perioperative variables in the two groups.

Groups	All (*n* = 150)	EAD (*n* = 53)	Non-EAD (*n* = 97)	*P* value
Preoperative data				
WBC (10^9^/L)	3.53 (2.32, 5.38)	3.72 (2.79, 6.60)	3.34 (2.18, 5.00)	0.021
NLR (%)	3.02 (1.66, 5.64)	2.92 (1.56, 5.55)	3.08 (1.97, 5.59)	0.749
PLT (10^9^/L)	66 (47, 106)	71 (55, 126)	63 (44, 96)	0.159
Albumin (g/L)	33.8 ± 7.0	33.7 ± 7.8	33.8 ± 6.5	0.945
TB (μmol/L)	40.8 (20.7, 147.2)	34.1 (19.8, 186.6)	41.0 (21.8, 114.2)	0.983
AST (U/L)	39 (28, 57)	42 (29, 70)	38 (28, 51)	0.275
ALT (U/L)	29 (20, 45)	33 (20, 54)	25 (20, 39)	0.089
SCr (μmol/L)	63.5 (53.4, 78.0)	62.0 (53.6, 79.8)	63.7 (53.2, 77.2)	0.841
PA (%)	63.8 (44.4, 77.9)	66.5 (43.9, 78.9)	62.8 (44.9, 75.6)	0.788
Ammonia (μmol/L)	71 (50, 94)	73 (51, 94)	68 (50, 94)	0.661
Intraoperative data				
Duration surgery (h)	9.26 ± 2.48	9.37 ± 2.82	8.93 ± 1.91	0.265
Duration of IVC interruption (min)	74 (58, 94)	78 (66, 97)	73 (57, 92)	0.140
Blood loss (L)	0.8 (0.5, 1.2)	0.8 (0.5, 1.5)	0.9 (0.5, 1.0)	0.226
PRBC transfusion (L)	0.8 (0, 1.6)	0.8 (0, 1.6)	0.8 (0, 1.6)	0.625
Urine output (ml/h)	178.1 (115.9, 261.6)	168.8 (103.8, 225.3)	187.3 (124.9, 268.9)	0.050
Fluid balance (L)	4.0 ± 1.8	4.4 ± 2.2	4.0 ± 1.6	0.200
Maximum dose of NE (μg/kg/min)	0.2 (0.1, 0.4)	0.5 (0.1, 0.5)	0.2 (0.1, 0.3)	0.061
Cold ischemic time (h)	7.7 ± 1.7	8.1 ± 1.8	7.5 ± 1.7	0.052
Warm ischemic time (min)	2.9 ± 0.7	2.9 ± 0.8	2.9 ± 0.7	0.936
Postoperative data^a^				
APACHE II score	14.8 ± 6.8	15.9 ± 8.0	14.2 ± 6.0	0.023
SOFA score	6.9 ± 3.8	7.8 ± 4.5	6.3 ± 3.3	0.140
Lactate (mmol/L)	1.9 (1.2, 3.0)	2.1 (1.5, 4.1)	1.6 (1.1, 2.6)	0.011
WBC (10^9^/L)	6.38 (4.12, 8.94)	7.08 (4.08, 9.52)	6.22 (4.19, 8.82)	0.684
NLR (%)	25.4 (15.1, 34.5)	25.5 (15.4, 30.4)	25.4 (16.0, 37.6)	0.466
PLT (10^9^/L)	57 (34, 84)	59 (35, 77)	56 (34, 85)	0.613
PA (%)	38.2 (28.9, 45.5)	33.1 (23.6, 42.1)	39.9 (31.4, 47.6)	0.002
Albumin (g/L)	33.1 ± 5.9	32.6 ± 6.6	33.4 ± 5.5	0.391
Mb (ng/ml)	871 (537, 1385)	1057 (639, 1740)	750 (509, 1065)	0.001
Ammonia (μmol/L)	61 (45, 85)	62 (46, 96)	60 (45, 76)	0.313
PCT (ng/ml)	20.2 (5.0, 39.8)	30.9 (8.5, 82.5)	14.2 (4.7, 28.6)	0.007
Vasoactive drug [*n* (%)]	64 (42.7)	28 (52.8)	36 (37.1)	0.063
Sepsis [*n* (%)]	90 (60.0)	36 (67.9)	54 (55.7)	0.143
Outcomes				
AKI [*n* (%)]	53 (35.3)	30 (56.6)	23 (23.7)	<0.001
ICU stay (days)	4.6 (2.8, 6.7)	5.6 (3.3, 10.4)	4.2 (2.7, 5.7)	0.003
Hospital stay (days)	44.7 (28.0, 68.1)	46.3 (31.3, 63.2)	44.1 (26.0, 69.2)	0.693
28-day death [*n* (%)]	11 (7.3)	7 (13.2)	4 (4.1)	0.041

Values are expressed as the mean ± SD, mean (IQR), or *n* (%).WBC, white blood cell; NLR, neutrophil to lymphocyte ratio; PLT, platelet; Scr, serum creatinine; AST, aspartate transaminase; ALT, alanine transaminase; TB, total bilirubin; PA, prothrombin activity; Mb, myoglobin; PCT, procalcitonin; PRBCs, packed red blood cells; IVC, inferior vena cava; NE, norepinephrine; SOFA, sequential organ failure assessment; APACHE II, acute physiology chronic health disease classification system II; AKI, acute kidney injury; ICU, intensive care unit; EAD, early allograft dysfunction.

^a^ Postoperative data were collected upon admission to ICU immediately.

### Predictive value of different models based on logistic analysis

In univariate logistic analysis, preoperative WBC, duration of IVC interruption, cold ischemic time, serum Mb on POD0, serum PCT, and PA may be related to the increased risk of EAD (*P *< 0.1). Based on multivariate logistic analysis, serum Mb on POD 0 [1.001 (1.000, 1.001), *P *= 0.02] and PCT on POD0 [1.008 (1.001, 1.015) *P *= 0.022] were independent risk factors for the incidence of EAD (Table 3).

**Table 3:** Univariate and multivariate logistic analyses of risk factors for EAD.

Variables	Univariate analysis			Multivariate analysis		
	OR	95% CI	*P* value	OR	95% CI	*P* value
Age (years)	1.008	0.962, 1.056	0.735			
BMI (kg/m^2^)	0.933	0.821, 1.060	0.284			
MELD score	0.960	0.903, 1.021	0.193			
WBC (10^9^/L)	1.215	1.030, 1.434	0.021			
ALT (U/L)	1.007	0.997, 1.017	0.195			
Duration of IVC interruption (min)	1.014	1.000, 1.028	0.058			
Urine output (ml/h)	1.000	0.995, 1.005	0.964			
Maximum dose of NE (μg/kg/min)	1.470	0.330, 6.550	0.613			
Cold ischemic time (h)	1.253	0.970, 1.618	0.084			
APACHE II score	0.994	0.925, 1.068	0.866			
Lactate (mmol/L)	1.153	0.951, 1.399	0.148			
Mb (ng/ml)	1.001	1.000, 1.001	0.041	1.001	1.000, 1.001	0.002
PCT (ng/ml)	1.007	0.999, 1.016	0.070	1.008	1.001, 1.015	0.022
PA (%)	0.960	0.921, 1.001	0.058			
Vasoactive drug	0.802	0.307, 2.094	0.652			

OR, odds ratio; 95% CI, 95% confidence interval; EAD, early allograft dysfunction; BMI, body mass index; MELD, model for end-stage liver disease; WBC, white blood cell; ALT, alanine transaminase; IVC, inferior vena cava; NE, norepinephrine; APACH II, acute physiology chronic health disease classification system II; Mb, myoglobin; PCT, procalcitonin; PA, prothrombin activity.

**Figure 2:**
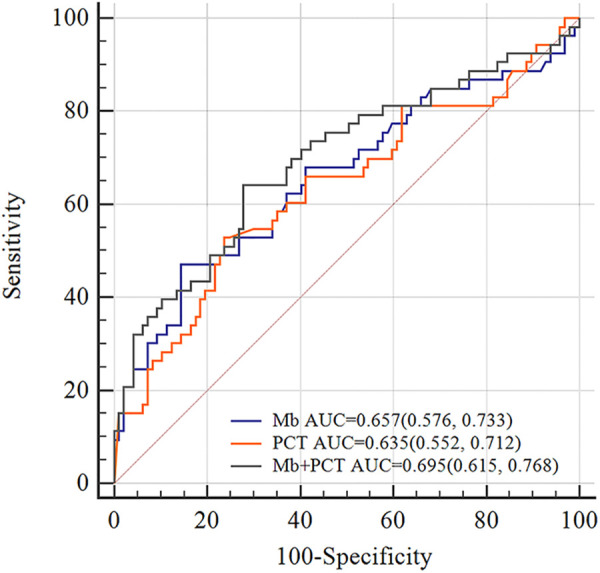
ROC curves of Mb, PCT, and Mb** + **PCT for EAD. The AUC values of Mb, PCT, and Mb + PCT were 0.657, 0.635, and 0.695, respectively. In total, 1327 ng/ml was the best cutoff value of myoglobin for predicting EAD (sensitivity = 47.2%, specificity = 85.6%). AUC, area under the curve.

Furthermore, we constructed different models to predict EAD based on independent risk factors. I predictive utility of Mb, PCT, and combined model for post-LT EAD is presented in Table 4. The AUC of serum Mb prediction was 0.657 (95% CI 0.561–0.754, sensitivity = 47.2%, specificity = 85.6%). Although Mb combined PCT to predict, the prediction was not good (AUC = 0.695, 95% CI 0.602–0.788, sensitivity = 62.4%, specificity = 72.2%). The ROC curve analysis is shown in Figure 2.

**Table 4:** Prediction of different models for EAD.

Models	AUC	SE	95% CI	Sensitivity	Specificity	*P* value
Mb (ng/ml)	0.657	0.049	0.561, 0.754	0.472	0.856	0.001
PCT (ng/ml)	0.635	0.050	0.537, 0.732	0.528	0.763	0.007
Mb + PCT*	0.695	0.047	0.602, 0.788	0.624	0.722	<0.001
Mb + PCT**	0.760	0.059	0.647, 0.874	0.806	0.607	<0.001
Mb + PCT***	0.800	0.073	0.648, 0.907	0.647	0.920	0.001

EAD, early allograft dysfunction; Mb, myoglobin; PCT, procalcitonin; AUC, area under the curve; SE, standard error; 95% CI, 95% confidence interval.

* All patients included in the analysis.

** Patients with preoperative MELD ≤ 15.

*** Patients with operative time ≥ 10 h.

### Dynamic changes of Mb with 3 days after LT

**Figure 3:**
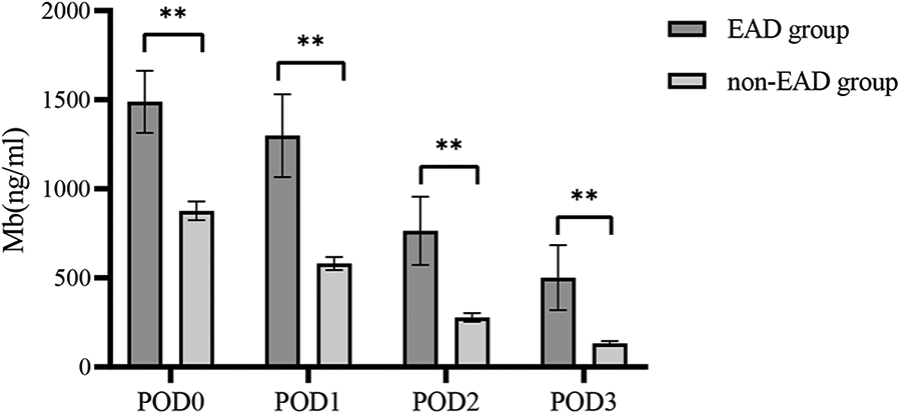
Distribution characteristics of myoglobin within 3 days between the EAD group and the non-EAD group after LT. The level of Mb decreased gradually after LT. Mb, myoglobin; EAD, early allograft dysfunction; POD, postoperative day; LT, liver transplantation. **P < 0.01 compared with the non-EAD group.

We examined changes in Mb and found that the level of Mb decreased gradually after LT, as shown in Figure 3. A significant difference could be discovered between the two groups for different periods within 3 days after LT (*P *< 0.05).

### Subgroup analysis

**Figure 4:**
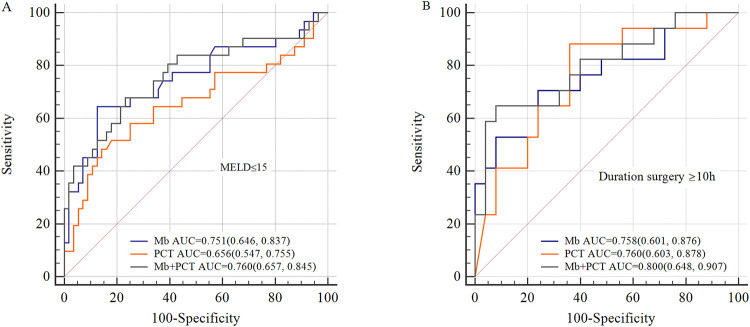
Subgroup analysis for ROC curves. (**A**) ROC curves of Mb, PCT, and Mb + PCT for EAD in patients whose preoperative MELD ≤ 15; (**B**) ROC curves of Mb, PCT, and Mb + PCT for EAD in patients whose operative time ≥ 10 h. The predictive value was better in patients whose preoperative MELD ≤ 15 or operative time ≥ 10 h (AUC = 0.751, 0.758, respectively, for Mb).

A previous study has identified MELD score, recipient age, and duration of surgery as risk factors for EAD. So, we performed a subgroup analysis based on these factors. In patients with preoperative MELD ≤ 15, the model of Mb, PCT, and Mb + PCT showed good prediction (AUC = 0.751, 0.656, 0.760, respectively), which was better in patients with duration of surgery ≥ 10 h (AUC = 0.758, 0.760, 0.800, respectively) (Figure 4).

### Relationship between Mb and duration of surgery

As shown in Supplementary Figure S1, we found that an increased duration of surgery (≥10 h) significantly increased the serum level of Mb (*P *= 0.003). However, no linear relationship existed between operative time and Mb. Also, comparing perioperative variables, operative time was not associated with EAD (*P *= 0.265).

## Discussion

Liver transplantation is the most effective treatment for patients with liver tumors or liver failure. With improved surgical techniques, the complications after LT have decreased significantly over the past decade. In our center, orthotopic liver transplantation (OLT) is mostly used, and the surgical technology is relatively mature. However, EAD is still a common complication after LT (2). Development of EAD after LT can impact short- and long-term prognosis. Thus, a better understanding of risk factors associated with EAD was critical for improving mortality.

Our current article demonstrated an independent association of the level of Mb on PDO0 with the incidence of EAD after LT. In our multivariate logistic analysis, we found that the risk of postoperative EAD was higher in patients with high Mb levels. However, its predictive value was limited. Accordingly, we performed subgroup analysis. For patients with preoperative MELD ≤ 15 or operative time ≥ 10 h, Mb could attain higher prediction. In addition, we observed a declined tendency of the Mb level from POD0 to POD3, and a statistical difference was also discovered between patients with EAD and those without EAD within 3 days after surgery. To our knowledge, the present study is the first to describe the predictive utility of postoperative Mb for EAD after LT.

The estimated incidence of EAD after LT varies in different reports (2, 19, 20). A study demonstrated that the incidence of EAD was 46.4% (21). However, in the present study, 35.3% of patients experienced EAD after LT. The major reason may be the drafts from DBD in our study. In addition, the occurrence of EAD can significantly result in poor outcomes and high mortality. In this study, patients with EAD experienced prolonged ICU stays and higher motility within 28 days. So, it is of significance to identify innovative clinical markers for early recognition of EAD. However, the etiology of EAD is still unclear and complex. IRI was mostly believed to lead to EAD after LT (6). IRI is associated with hypoxia of liver cells, sinusoidal endothelial cell damage, or the release of inflammatory factors (4, 20, 22).

A number of risk factors for the incidence of EAD have been identified by previous studies including recipient-related, donor-related, and surgical factors (7, 9, 23). Golse et al. reported the potentially predictive utility of lactate for EAD. They indicated that arterial lactate concentration at the end of LT, as a reflection of tissue perfusion, was a strong predictor of EAD (24). A similar result was observed in our study, but serum lactate was not a predictor of EAD. The possible reason is that lactate was acquired immediately after admission to ICU. Kuse et al. demonstrated that PCT, which is mainly produced by the liver, allowed differentiation between infection and rejection after LT (25). Our study found that PCT was associated with a higher risk of EAD. Also, previous studies have identified that PCT could reflect the severity of liver injury (26). In addition to these risk factors, some other variables, to our knowledge, were also the risk factors for the incidence of EAD. Quite a few studies have confirmed that donor age over 60 years, donor BMI, graft steatosis, predictive serum sodium, cold ischemia time, and warm ischemia time were important risk factors for EAD after LT (5, 27–29).

Myoglobin is found in skeletal muscle and heart muscle cells and will be released into the blood when a muscle is damaged. During LT, muscle damage occurs by prolonged postural retention and intraoperative muscle pulling, and when rhabdomyolysis occurs, Mb is released into the blood (30). During liver transplantation, the prolonged operative time means longer immobilization or certain surgical positions, which will lead to varying degrees of muscle damage. In addition, due to the presence of anhepatic phase and IRI during LT in all patients, muscle cell injury also results from insufficient blood supply and microcirculatory disturbance (31). In our current study, we demonstrated that prolonged operative time leads to higher postoperative serum Mb. The reason is that this phenomenon may result from more serious muscle damage in prolonged surgery.

In the previous study performed by Fricke et al., they indicated that the level of Mb could predict the extent of impairment after revascularization (32). When Mb was more than 20,000 µg/L, there was an obvious correlation with organ failure. Our results also showed that an increased serum Mb level was closely related to the incidence of EAD after LT. Moreover, the elevated Mb may be related to an inflammatory reaction, and a previous study indicated that elevated Mb is associated with sepsis (33). Before our study, there was no study on the relation between Mb and the occurrence of EAD. Thus, the pathophysiological of Mb predicting utility for EAD after LT was unclear. A previous study discovered that the level of serum Mb peaked a few hours after reperfusion during surgery (32), and quick reperfusion may help reduce Mb release into the bloodstream. Clinically, serum myoglobin can be partially removed by continuous renal replacement therapy and hemoperfusion. Our findings may provide a new direction for treating EAD in patients who underwent LT. In addition, we believed that the level of Mb might reflect the severity of inflammatory responses, which play an important role in the occurrence of EAD after LT. Further study of animal experiments is still needed to explore the pathophysiological mechanism.

Our innovative study is the first to discover the association between postoperative Mb and the risk of EAD. Despite being novel, there are a few limitations to this study. First, it was limited due to a single center and department with a small sample size. Second, although one person finished data collection, there was still bias. Finally, as the study was performed in a single department, which was the surgical ICU, the variables related to donors that may influence the occurrence of EAD were difficult to obtain.

## Conclusion

Among patients after LT, postoperative Mb gradually decreased within 3 days after surgery. Mb might be a novel predictor of EAD. In addition, the predictive value of Mb combined PCT was better, especially in patients with preoperative MELD ≤ 15 or operative time ≥ 10 h.

## Data Availability

The raw data supporting the conclusions of this article will be made available by the authors, without undue reservation.
