# Supplementary material for: Postoperative serum myoglobin as a predictor of early allograft dysfunction after liver transplantation
Source: Front Surg. 2022 Oct 12;9:1026586. doi: 10.3389/fsurg.2022.1026586 (PMC9597078; doi:10.3389/fsurg.2022.1026586)
Supplement: Supplementary file 1 [file Table2.docx]

**Supplement meterials**

Title: Postoperative serum myoglobin as a predictor for early allograft dysfunction after liver transplantation

**Table S1: KDIGO criteria of AKI for adult patients**

**Table S2:** **The relationship of operative time and serum Mb.**

**Table S1.** KDIGO criteria of AKI for adult patients

| **Stage** | **Serum creatinine** | **Urine output** |
| --- | --- | --- |
| 1 | 1.5-1.9 times baseline OR  ≥0.3 mg/dl (≥26.5 μmol/l) increase | <0.5 ml/kg/h for 6-12 hours |
| 2 | 2.0-2.9 times baseline | <0.5 ml/kg/h for ≥12 hours |
| 3 | 3.0 times baseline OR  Increase in serum creatinine to ≥4.0 mg/dl (353.6 μmol/l) OR  Initiation of renal replacement therapy OR,  in patients <18 years, decrease in eGFR to 35 ml/min per 1.73 m^2^ | <0.3 ml/kg/h for ≥24 hours OR  Anuria for ≥12 |

Abbreviations: KDIGO, Kidney Disease Improving Global Outcome; AKI, Acute Kidney Injury.


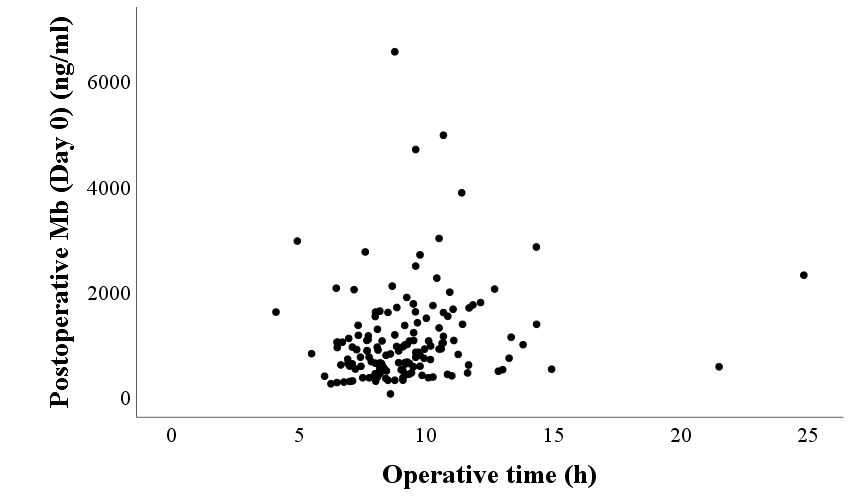

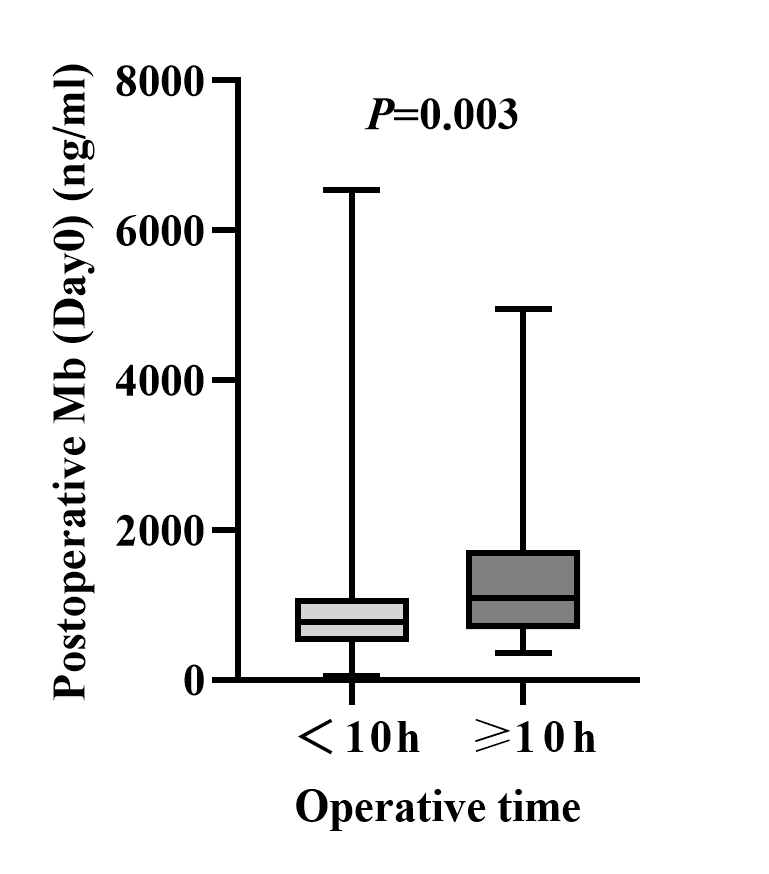


**Figure S1. The relationship of operative time and serum Mb.**

Abbreviations: Mb, myoglobin. There was significant difference between Mb and operative time (*P*=0.003) even though on linear relationship existed between the two variables.
